# Wnt/β-catenin signaling pathway in the tumor progression of adrenocortical carcinoma

**DOI:** 10.3389/fendo.2023.1260701

**Published:** 2024-01-09

**Authors:** Yanghao Tai, Jiwen Shang

**Affiliations:** ^1^ Third Hospital of Shanxi Medical University, Shanxi Bethune Hospital, Shanxi Academy of Medical Sciences Tongji Shanxi Hospital, Taiyuan, China; ^2^ Department of Ambulatory Surgery, Shanxi Bethune Hospital, Shanxi Academy of Medical Science, Tongji Shanxi Hospital, Third Hospital of Shanxi Medical University, Taiyuan, China

**Keywords:** adrenocortical carcinoma, Wnt/beta-catenin, therapeutic targets, tumor progression, cross talk

## Abstract

Adrenocortical carcinoma (ACC) is an uncommon, aggressive endocrine malignancy with a high rate of recurrence, a poor prognosis, and a propensity for metastasis. Currently, only mitotane has received certification from both the US Food and Drug Administration (FDA) and the European Medicines Agency for the therapy of advanced ACC. However, treatment in the advanced periods of the disorders is ineffective and has serious adverse consequences. Completely surgical excision is the only cure but has failed to effectively improve the survival of advanced patients. The aberrantly activated Wnt/β-catenin pathway is one of the catalysts for adrenocortical carcinogenesis. Research has concentrated on identifying methods that can prevent the stimulation of the Wnt/β-catenin pathway and are safe and advantageous for patients in view of the absence of effective treatments and the frequent alteration of the Wnt/β-catenin pathway in ACC. Comprehending the complex connection between the development of ACC and Wnt/β-catenin signaling is essential for accurate pharmacological targets. In this review, we summarize the potential targets between adrenocortical carcinoma and the Wnt/β-catenin signaling pathway. We analyze the relevant targets of drugs or inhibitors that act on the Wnt pathway. Finally, we provide new insights into how drugs or inhibitors may improve the treatment of ACC.

## 1 Introduction

Adrenocortical carcinoma (ACC) is an uncommon, aggressive endocrine malignancy originating from the adrenal gland, influencing 0.5 to 2 persons/million individuals annually worldwide (1, 2). A 5-year survival rate of about 35% following diagnosis, dropping to only 13-16% for stage IV patients, and a significant risk of recurrence and metastasis are all indicators of its typically dismal prognosis (3–5). Currently, only Mitotane received certification from both the US Food and Drug Administration (FDA) and the European Medicines Agency for the medication of advanced ACC. However, it has minimal therapeutic efficiency and hazardous side effects in the advanced stages of the disease (6, 7). Despite advances in other treatment options, the survival rate of patients with ACC has not altered over the past 40 years (8). Various Wnt signaling inhibitors, acting on different targets, have been discovered. Many of them have shown effective and potential roles in anti-cancer. However, up to now, there are no Wnt inhibitors approved for the treatment of ACC. Previous literature reviews only described the major findings about the relationship between ACC and the Wnt/β-catenin signaling pathway. There is still a gap in the comprehensive description of drugs with relevant potential target effects.

Based on the relationship between ACC and Wnt signaling, our study summarizes the main findings of biological mechanisms. At the same time, we first describe the new drugs that act on the Wnt signaling and its relative inhibitors, providing new insights into how drugs or inhibitors may improve the treatment of ACC.

## 2 Overview of the WNT signaling pathway

**Figure 1:**
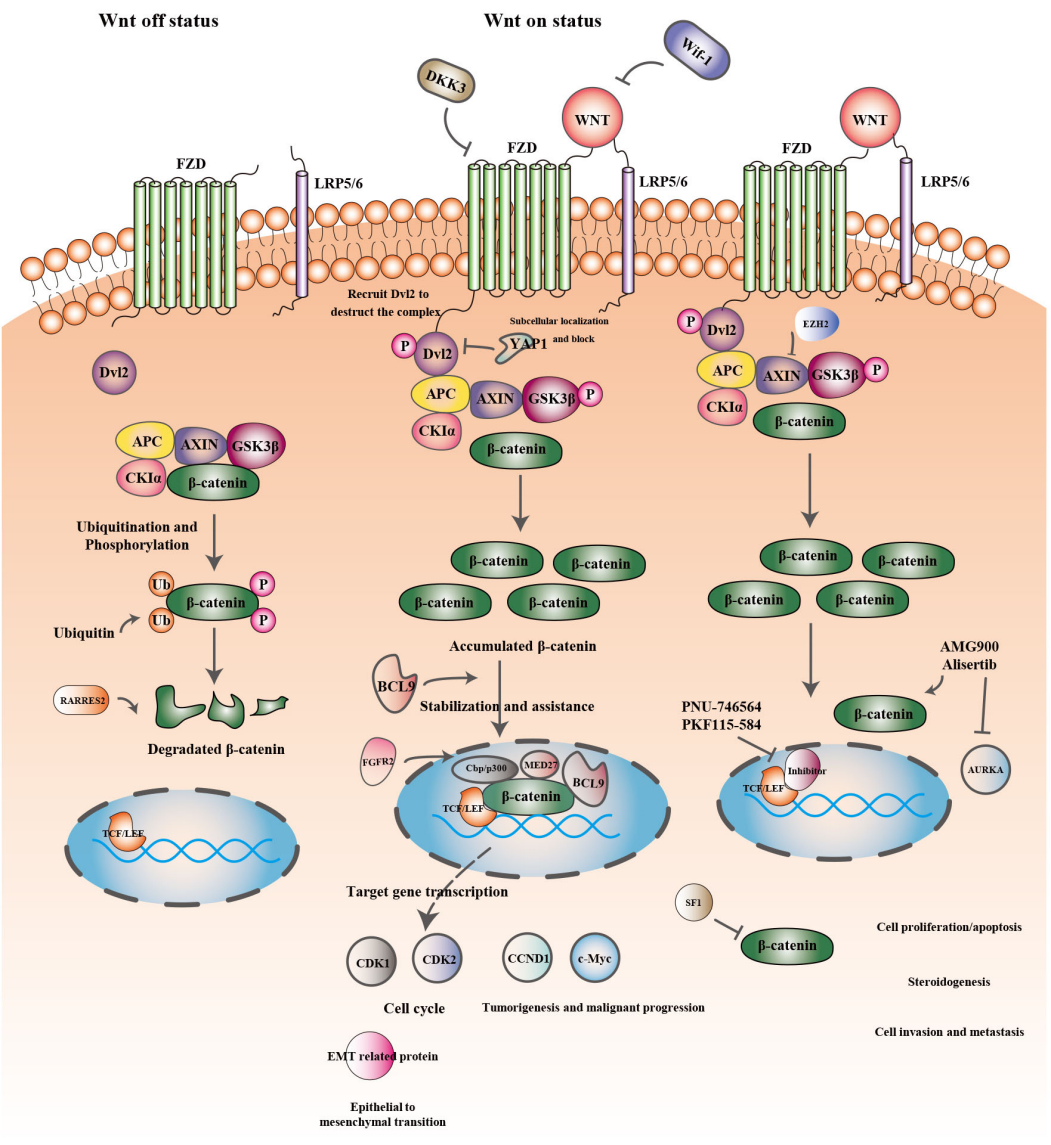
Wnt/β-catenin signaling pathway and crosstalk involved in this review.

The Wnt signaling pathway is one of the evolutionarily conserved signaling pathways that control a variety of physiological processes, including cellular apoptosis, proliferation, cellular polarity fate, determination, stem cell maintenance, and migration during development (31, 32). The key factor in the emergence and development of several tumors is the dysregulation of Wnt signaling (33–35). The signaling cascade consists of different branches: the Wnt/β-catenin or canonical Wnt signaling pathway, the Wnt/Ca^2+^ signaling pathway, and the planar cell polarization (Wnt-PCP) pathway. Recently conducted studies have concentrated on the Wnt/β-catenin signaling pathway, which is involved in the emergence of several diseases (36). **Table 1** showed the Overview of the WNT signaling pathway. **Figure 1** showed the Wnt/β-catenin signaling pathway and crosstalk involved in this review.

**Table 1:** The overview of this review.

No.	Categorizations	Expression	Functions	References
1	Overview of the WNT signaling pathway			
1.1	Canonical Wnt/β-catenin signaling pathway			
1.2	Non-canonical pathways			
2	Alterations in Wnt			
2.1	Wnt ligands	Overexpressed	Activation of Wnt/β-catenin pathway	(9, 10)
2.2	Wif1	Downregulated	Activation of Wnt/β-catenin pathway	(11)
2.3	DKK3/FOXO1	Downregulated	Cell motility and clonal development	(12–14)
2.4	β-catenin	Transcriptionally active	Cell proliferation and apoptosis	(15–17)
3	Transcription factor regulation			
3.1	BCL9	Overexpressed	Tumor progression	(18)
3.2	YAP1	Overexpressed	Cell migration and cell viability	(19)
3.3	AURKA	Overexpressed	Cell proliferation, viability, invasion, and cortisol release	(20, 21)
3.4	MED27	Overexpressed	Cell proliferation, invasion, apoptosis, and cycle	(22)
4	Growth factor signaling			
4.1	FGFR2	Overexpressed	β-catenin phosphorylation and response to WNT protein	(23, 24)
4.2	IGF2	Overexpressed	Cell proliferation and viability	(25)
4.3	SF-1	Overexpressed	Inhibition of Wnt/β-catenin pathway	(26)
5	Epigenetic regulation			
5.1	EZH2	Overexpressed	Cell viability, clonal expansion, and apoptosis	(27, 28)
5.2	AFF3	Overexpressed	Inhibition of Wnt/β-catenin pathway	(29)
5.3	RARRES2	Downregulated	Cell proliferation and cell invasion	(30)
6	Drugs and inhibitors			
7	Conclusion and prospect			

### 2.1 Canonical Wnt/β-catenin signaling pathway

For Wnt/β-catenin signaling to occur, the Wnt ligand must attach to its coreceptor complex, which is composed of the Frizzled (FZD) protein family and low-density lipoprotein receptor-related protein 5 (LRP5) or LRP6 (37). Casein kinase I(CKI), glycogen synthase kinase 3 (GSK3β), Adenomatous polyposis coli (APC), and Axin form a complex that phosphorylates β-catenin located in the cytoplasm in the absence of Wnt ligands. In this instance, Axin supports the formation of a complex with GSK3β and APC (38–41). Once the complex is formed, GSK3β promotes the phosphorylation of cytoplasm β-catenin, and APC facilitates the combination of the ubiquitin-mediated protein hydrolysis pathway to phosphorylated β-catenin in the cytoplasm. When Wnt ligands are present, they attach to their coreceptor complex and then trigger the Wnt signaling by enlisting Dvl proteins in the cytoplasm and preventing or interrupting the fabrication of the Axin/GSK3/APC complex. This prevents β-catenin from being degraded and causes it to build up in the cytoplasm. The Accumulated proteins translocate into the nucleus, combining with T-cell Factor/Lymphoid Enhancing Factor 1 (TCF/LEF1) thereby regulating the specified genes’ transcription (42–44). In addition, the Wnt/β-catenin signaling pathway interacts with multiple other pathways. In gastrointestinal and breast cancers, it synergizes with TGFβ to enhance fibrosis and EMT (epithelial-mesenchymal transition) at the transcriptional level (45, 46). Other research demonstrated that the Hippo and Notch signaling networks can interact with the Wnt/β-catenin pathway. Several malignancies, such as endometrial carcinoma, hepatocellular carcinoma, and adrenocortical carcinoma, involve CTNNB1 genetic mutations that encode β-catenin (47–49). Phosphorylation sites necessary for the degradation of β-catenin function as mutational hotspots, leading to β-catenin translocation and accumulation to the nucleus, which in turn regulates genetic transcription (50).

### 2.2 Non-canonical pathways

The homeostasis of both adult and embryonic tissues is connected to the non-canonical planar cell polarity (PCP) pathway, uninvolved by the co-receptor LRP5 or β-catenin. The pathway is initiated through the interaction of Wnt with co-receptors such as ROR2 (receptor tyrosine kinase-like orphan receptor 2), Ryk (RYK receptor-like tyrosine kinase), and FZD and then subsequently triggers the recruitment of protein Dvl to activate c-Jun-N-terminal kinase (JNK) and/or Rho family GTPases (51, 52). The Wnt/Ca^2+^ pathway is an extra non-canonical pathway that also disregards β-catenin.

The interaction of Wnt ligands with FZD leads to the transient escalation of Ca^2+^ concentration and then increases the production of inositol 1,4,5-trisphosphate under the condition of activated PLC (phospholipase C). The interaction between IP3 and calcium channels located on the surface of the endoplasmic reticulum causes the elevation of Ca^2+^ concentration and the activated CaMKII (Calcium-CaM-dependent protein kinase II). Several regulatory proteins, such as NF-κB, CREB, and NFAT, are activated by the Ca^2+^-PLC pathway (53). Further research is required to confirm the reports that FYN-STAT and YAP-TAZ are connected to the non-canonical Wnt pathway (54–56). In addition, sFRP (secreted Fzd-related proteins), Dickkopf family (Dkks), and WIFs (Wnt inhibitory factors) can antagonistically affect tumorigenesis and development mediated by the Wnt pathway (33).

## 3 Alterations in Wnt

### 3.1 Wnt ligands

WNT proteins are a class of cysteine-rich secreted glycoprotein signaling molecules. They are involved in tumor development through biological processes such as cell proliferation, apoptosis, migration, and differentiation. According to different biological functions, they are classified into two categories, non-canonical signaling substances and canonical WNT/β-catenin signaling ones (57, 58). High expression of Wnt4 was detected in primary adrenocortical carcinoma cells and tissues (9). Bioinformatic analysis revealed that in ACC tumor tissues, overexpressed Wnt5A was associated with poorer prognostic survival, including progression-free interval (PFI), disease-specific survival (DSS), and overall survival (OS). In ACC, Wnt5A overexpression was positively correlated with microsatellite instability and tumor mutational load, suggesting that it may be a prognostic marker for immunosuppressive checkpoints (10).

### 3.2 Wif1

In kidney and bladder tumors, dysregulation of Wnt antagonists has been identified as an alternate mechanism for the abnormally activated Wnt signaling pathway (59, 60). Promoter CpG methylation leads to the downregulation of Wif-1 in adrenocortical tumors. The epigenetic dysregulation might activate the Wnt/β-catenin pathway, which would then stimulate downstream target gene CCND1 expression to participate in tumorigenesis (11). However, concrete proof is scarce for the mechanism of Wif-1 regulation in adrenocortical carcinoma.

### 3.3 DKK3/FOXO1

A 38 kDa secreted glycoprotein called dickkopf-associated protein 3 (DKK3), with a signaling peptide at its N-terminal, is dependent on co-expressed ligands and cell surface receptors to exert inhibitory effects in Wnt signaling (61, 62). The level of DKK3 expression is low in the majority of solid tumors and mediates cell apoptosis and/or cycle arrest in over-expression research of various cancer cell types (63–66), exerting a tumor-suppressive role of Wnt signaling regulators. Additionally, ectopic expression of DKK3 suppresses malignant invasion and migration and reverses EMT effects in multiple cancer cell types, indicating that DKK3 also has a dedifferentiation-blocking function (67, 68). DKK3 is weakly expressed in most adrenocortical carcinoma tumor tissues (12), suggesting a possible oncogenic role in ACC. However, no correlation between clinicopathological features such as age, sex, ENSAT stage, size and weight of the tumor, hormone-secreting phenotype, and expression levels has been observed to correlate significantly (12, 69). Epigenetic modifications, such as chromatin condensation and promoter methylation, are both the mechanisms of DKK3 silencing in the majority of other cancers (66). According to Joyce Y Cheng et al, promoter hypermethylation may contribute to the suppression of DKK3 expression in adrenocortical carcinoma (12). Gene copy number variations have reportedly been linked to adrenocortical carcinogenesis (70, 71). Gene copy loss downregulates DKK3 expression in most ACC samples. However, only a small percentage of these samples concurrently had promoter methylation. This implies that gene copy loss can downregulate DKK3 expression independently from promoter methylation. Furthermore, in ACC tumorigenesis, copy number alterations might manifest more precious than gene-specific methylation (12). Due to the mutations of CTNNB1 and AXIN2, constitutively active Wnt/β-catenin signaling is generated in the NCI-H295R cell line. This cell type is unaffected by DKK3 partial silencing or exogenous recombination in terms of viability, clonal growth, or migration, possibly due to the resistance generated by constitutively activated Wnt signaling. The endogenous DKK3 is expressed by the SW13 cell line. Silencing DKK3 expression promotes cell motility and inhibits tumor clonal development, but has little effect on cell viability (13, 14). Exogenous DKK3, in contrast, promotes migration, proposing that endogenous and secreted DKK3 represent distinct functions and may have cellular signaling targets distinct from the traditional Wnt/β-catenin transmission (61). Additionally, constitutive overexpression of DKK3 prevents the clonal expansion and invasive activity of ACC cells, maybe because of the morphologically differentiated lobular pseudopods’ increased attachment to the stroma (72–74). The function of DKK3 in promoting the ACC cell redifferentiation phenotype and/or anti-invasive signaling is partially mediated through FOXO1 (12). FOXO1 is discovered as a potential downstream target of the TGF-β signaling pathway, which also contributes to the pathophysiology of ACC. FOXO1 is weakly expressed in adrenocortical carcinomas. *In vitro*, silencing of FOXO1 resulted in apoptosis-mediated suppression of viability in SW13 cells along with enhanced cell migration behavior. These findings point to a specific function for FOXO1 in controlling the vitality and motility of adrenocortical cells (12, 75). The tumor-suppressive effect of viability inhibition due to gene downregulation contradicts the enhanced migratory behavior, which may be related to the complex signaling crosstalk in SW13 cells. The enhanced migratory behavior may be due to the involvement of FOXO1 in Wnt signaling-mediated motility restriction, which needs to be verified by separate downstream signaling experiments. Summarily, the research of DKK3/FOXO1 signaling in the adrenal cortex may contribute to the generation of novel medications with a focus on re-differentiation.

### 3.4 β-catenin

Adrenocortical carcinogenesis is fueled by the abnormally active Wnt/β-catenin signaling pathway (76), whose vital component is β-catenin and researchers have demonstrated a substantial association between the extent of β-catenin nucleus staining and higher Weiss scores, greater ENSAT tumor stage (stage III and IV), CTNNB1/APC mutations, more frequent mitosis and necrosis, and as well as with poorer OS and PFI in patients (49). In adrenocortical carcinoma, aberrant β-catenin status correlates with upregulation of its target genes LEF1, AXIN2, and ISM1, which are not increased in ACA, indicating that transcriptionally active β-catenin influences proliferative phenotype and the transcriptional level of TCF/LEF target genes (15, 16). Silencing CTNNB1 inhibits H295R cell proliferation and stimulates apoptosis by reducing Wnt/β-catenin-LEF/TCF-dependent transcription (15). Additionally, as a Wnt/β-catenin pathway antagonist, PNU-74654 (PNU) functions through competitively binding TCF to interfere with protein-protein interactions. It has been demonstrated that PNU promotes apoptosis and prevents proliferation by blocking the TCF/β-catenin complex (15). Another study revealed that PKF115-584 dose-dependently promotes NCI-H295R cell apoptosis and also suppresses cell proliferation and β-catenin-dependent transcription (17). The primary cause of dysregulated cell proliferation is abnormal cell cycle progression. Different cell cycle inhibitors and proteins work together to regulate the cell cycle. CCND1, CDK1, and CDK2 are significant proteins that regulate the G1/S transition of the cellular cycle and are also repressed as downstream targets of Wnt signaling according to the aforementioned mechanism study (77). The same regulatory phenotype was observed for silencing CTNNB1 in a xenograft mouse model (16). The abnormally activated Wnt/β-catenin pathway in the adrenal cortex of the mouse models alone results in tissue hyperplasia. A malignant phenotype occurs in the adrenal cortex when p53 is simultaneously deleted (78).

## 4 Transcription factor regulation

### 4.1 BCL9

As a transcriptional co-activator of Wnt/β-catenin signaling, the oncogenic gene B-cell lymphoma 9 (BCL9) is essential for the formation and progression of a variety of malignancies (79). Targeted disruption of the BCL9/β-catenin complex inhibits oncogenic Wnt signaling (80, 81). Current studies have observed the association between the overexpression of BCL9 and tumor formation, including breast cancer, renal cell carcinoma, hepatocellular carcinoma, and colorectal cancer (82–85). BCL9 is elevated in adrenal malignancies, and its upregulation level is significantly associated with tumor aggressiveness. Immunohistochemical techniques revealed higher expression status in ACC tumor tissues with the distinct cytoplasm and nucleus diffuse expression pattern (18). Prior research has shown that BCL9 enhances tumor cell proliferation *in vitro (* 83). Silencing BCL9 expression significantly inhibited the clonal growth of SW13 cells. But in H295R cells which have the CTNNB1 mutation, silencing BCL9 did not interfere with the potential for clonal growth. This phenomenon indicates that high expression of BCL9 may accelerate ACC tumor progression by triggering the Wnt tumorigenic pathway (18). Earlier research has revealed the potential functions of BCL9 in tumor metastasis and invasion in colorectal cancer (85, 86) and significant upregulation was observed in ACC. Only 5.8% of the ACA cohort revealed more than a double increase of BCL9 expression levels, while 40 percents of the ACA tissues displayed a double expression upregulation, indicating that upregulation of BCL9 expression in adrenocortical carcinoma is correlated to the malignant characters. Taylor C Brown et al. attempted to identify the relationship between the different clinical characteristics and BCL9 expression patterns. However, no significant correlation was found, but there was a propensity towards elevated expression status for elderly individuals, although not reaching significance (18), which may be due to the limited cohort sample. The activity and/or stability of both molecules may be enhanced by the capacity of BCL9 to connect with the β-catenin, while the overexpression of β-catenin may have this same benefit (87). Regarded as the co-activator of β-catenin located in the nucleus, BCL9 can translocate β-catenin to the TCF and promotes the activation of Wnt-responsive transcription genes (cyclinD1, c-Myc), several of which are strongly associated with carcinogenesis and the progression of malignancy (88). Currently, a growing quantity of research initiatives have concentrated on medications that are protein-protein interaction inhibitors that disrupt interactions between β-catenin and Bcl9 in the tumor Wnt/β-catenin pathway in an attempt to uncover promising candidates for enhancing immunity and inhibiting tumor growth (89).

### 4.2 YAP1

As the Hippo pathway-associated transcription factor-like protein, Yes-associated protein1 (YAP1) is an oncogenic gene and it is involved in tissue regeneration, cell embryogenesis, and proliferation (90–92). In cancer cell lines, overexpressed YAP1 is correlated with the formation and growth of tumors. Furthermore, YAP1 can engage with multiple signaling pathways including Wnt/β-catenin, Notch, and Sonic Hedgehog (SHH), in addition to the Hippo pathway (91, 93, 94). For instance, YAP1 synergizes with β-catenin to activate genes necessary for epithelial repair and stem cell proliferation (90). Previous research has demonstrated that YAP1 can participate in inhibiting Wnt/β-catenin signaling by regulating the subcellular localization of DVL2 or blocking DVL2 (94–96). Immunofluorescence reveals overexpressed YAP1 both in the adrenocortical tumors (ACTs) of children, as well as in the cytoplasm and nucleus of fetal adrenal cells, while diminished expression of YAP1 is observed in the postnatal adrenal cortex, pointing to the potential involvement of YAP1 in promoting tissue dedifferentiation and proliferation (19). Treatment of the NCI-H295R cell lines with a TCF/β-catenin complex inhibitor (PNU-74654) observed a lessened protein expression but an increased mRNA expression. This phenomenon can be attributed to post-transcriptional regulation. The decrease in protein expression may result in negative feedback triggering an increase in mRNA expression. *In vitro* experiments involving the silencing of YAP1 demonstrated an increase in CTNNB1 nucleus and protein expression, without any noticeable alteration in Dishveld2 (DVL2) mRNA expression. This observation can be attributed to the ability of YAP1 to either sequester DVL2 in the cytoplasm or facilitate its translocation to the nucleus, depending on the β-catenin phosphorylation status (15, 19). In response to alterations in the different extracellular matrix (ECM), YAP1 participates in cellular mechanotransduction by interacting with cell adhesion molecule-bound α-catenin. In the hard ECM, activated YAP1 accumulates in the nucleus. Conversely, in softened ECM, YAP1 is accumulated and degraded in the cytoplasm (97–99). Additionally, increased ECM stiffness causes a deficiency of intercellular connections, which promotes metastasis and epithelial-mesenchymal transition (EMT) (100). Loss of intercellular junctions during EMT can block Hippo signaling and thus activate YAP1 (97). In the NCI-H295 cell line, the knockdown of YAP1 inhibited cell migration and cell viability, implying that YAP1 contributes to adrenocortical cell growth and metastasis. Furthermore, the mRNA expression of YAP1 was upregulated in patients with recurrence and/or metastasis (R/M) and death. The overexpression was correlated with worse OS of patients (19). These results highlight the correlation of YAP1 in relapsed and/or metastatic disease.

### 4.3 AURKA

The protein Aurora kinase (AURK) regulates the cell cycle and controls cell growth through involvement in DNA damage and kinase overexpression, with three subunits involved in cell division in the G1-M phase (101). In comparison to normal adrenal tissues, AURKA and AURKB expression was upregulated in adrenocortical carcinoma and three cell lines (CU-ACC1, CU-AAC2, and NCI-H295R), while no discernible differences were observed for AURKC. In both pediatric and adult patients, the over-expression of AURKA and AURKB was correlated with a worse prognosis, implying that kinases may be implicated in the tumorigenic effects of ACC (20). AMG900 is a highly selective and orally bioavailable pan-aurora kinase inhibitor that effectively reduces cell proliferation and is effective against multi-drug resistant cell lines. Treatment of the NCI-H295R cell line with AMG900 alone reduced cell viability, promoted apoptosis, and suppressed cell invasion and metastatic capacity and also inhibited cell proliferation, increased the chemosensitivity of the NCI-H295R cell lines to a variety of drugs including mitotane, doxorubicin and etoposide, among other anticancer drugs (20, 21). The considerable increase of CTNNB1, MYC, and c-MYC was observed after the application of the NCI-H295R cell lines with AMG900, suggesting that AMG900 may contribute to activating the Wnt/β-catenin pathway (20). The combination with PNU-74654, the Wnt/β-catenin signaling pathway inhibitor, had a greater impact on the suppression of cellular proliferation and viability, indicating that enhanced expression of c-Myc and CTNNB1 resulting in AMG900 treatment could be interdicted by PNU-74654, thus resulting in a synergistic antitumor effect. The inhibition of Aurora kinase caused by AMG900 prevented colony production and cell invasion in NCI-H295R cells, and the combination with PNU-74654 did not enhance this effect. Conversely, blocking the Wnt/β-catenin pathway had a better impact on reducing cortisol release from NCI-H295R compared to inhibiting Aurora kinase. The AURKA inhibitor Alisertib demonstrated good efficacy in phase I/II/III clinical trials and several tumor types. Compared to the combination of PNU-74654 and AMG900, the impact on, the combination of PNU-74654 and Alisertib was observed more effective in suppressing the cell viability of NCI-H295R cells, implying that the function generated by AMG900 on cell viability of adrenocortical carcinoma may be caused by the inhibition of AURKA. According to these studies, targeting ACC malignancies may be accomplished by inhibiting aurora kinase activity and blocking the β-catenin pathway simultaneously (20, 102).

### 4.4 MED27

The MED complex is a family of transcriptional co-activators consisting of multiple proteins that can participate in the regulatory process of genes dependent on RNA polymerase II transcription by interacting with transcription factors to turn on the assembly of transcription initiation complexes and consequently gene transcription (103–106). Overexpressed MED27 in ACC tissues is associated with low survival rates in patients. *In vitro* cellular and *in vivo* mouse models, silencing of MED27 decreases proliferation and cell invasion and induces apoptosis and cellular cycle organization. Additionally, suppressed MED27 resulted in altered expression levels of EMT-related proteins, suggesting that MED27 may mediate ACC invasiveness by stimulating the EMT procedure. Hongchao He et al. discovered that downregulated transcription of β-catenin and its target gene was observed by the knockdown of MED27, suggesting that Wnt/β-catenin pathways might contribute to the phenotypic regulation mediated by MED in ACC (22).

## 5 Growth factor signaling

### 5.1 FGFR2

Different fibroblast growth factors can stimulate the proliferation and expansion of adrenocortical cells (107–109). As a tyrosine kinase receptor, FGFR2 (Fibroblast growth factor receptor type 2) consisting of an intracellular tyrosine kinase structural domain and an extracellular immunoglobulin-like structural domain is encoded on human chromosome 10q26 (110, 111). The basic fibroblast growth factor-regulated transcriptional co-activator CITED2 (Cbp/p300 interaction trans-activator 2) participates in adrenal development in adrenocortical cells (23). Research has revealed that FGFR2 is associated with the growth and development of adrenal glands in mice. Absence of this receptor subtype leads to impaired adrenal differentiation and growth during the development of adrenal (112). Adrenocortical progenitor cells are stimulated to proliferate and prevent apoptosis by FGFR2 signaling (113). Current studies identified abnormal FGFR2 signaling as an essential factor in carcinogenesis and a possible therapeutic approach for various tumor types (114). The activated WNT/β-catenin pathway is one of the main mechanisms involved in the pathophysiology of adrenocortical carcinoma. Approximately 10 to 15 percent of patients have CTNNB1 activating mutations, which lead to aberrant nucleus accumulation of β-catenin (115). While FGFR signaling has been determined to trigger the canonic WNT signaling through β-catenin phosphorylation and increased cellular response to Wnt in other cell types (24),. The majority of adrenocortical carcinomas had diverse degrees of FGFR2 expression in the cytoplasm and nucleus, according to Matthias Haase et al. However, due to insufficient sample size, no significant correlation was discovered between CTNNB1 mutation status and other clinical characteristics (116). Alternatively, FGFR signaling may promote adrenocortical tumors by triggering WNT signaling upstream of growth, independent of CTNNB1 activating mutations. Additionally, there are two separate isoforms of FGFR2, namely FGFR2b (epithelial variation) and FGFR2c (mesenchymal variant), which differ in their immunoglobulin-like structural domain. Variable expression and splicing of FGFR2 isoforms may promote tumor progression under the mechanism of EMT (117). Therefore, further experiments might concentrate on the differential expression and intracellular localization of FGFR2 isoforms in adrenocortical cancer cells.

### 5.2 IGF2

As a key growth factor for adrenocortical growth, insulin-like growth factor 2 (IGF2) (117), acts as a mitogen binding with cell surface receptor IGF-1R, auto-phosphorylates and activates downstream signaling cascades and participates in processes such as cellular proliferation, and the regulation of cell cycle (118). IGF2 expression is upregulated in ACC, and *in vitro*, high concentrations of IGF2 promote H295R cell proliferation and increase cell viability while having no effect on invasive capacity (25). The overexpression of IGF2 is normally correlated to constitutive activation of β-catenin in ACC patients, implying that variations in both signaling pathways might expedite the development of malignancy. However, in the IGF2 transgenic adrenal tissues, Coralie Drelon et al. failed to detect activated Wnt/β-catenin signaling (119). Provided that Wnt signaling is activated, overexpressed IGF2 moderately accelerates cancer development but is insufficient to initiate the progression of malignant tumors (120).

### 5.3 SF-1

Steroid growth factor 1 (SF-1) is a nuclear receptor and it is involved in the expression of cell cholesterol homeostasis genes and steroid hormone synthesis (121) and also involved in the growth and development of steroid-producing glands such as adrenal and gonadal tissues (122, 123). By interacting with particular response components in target gene promoters, SF-1 recruits repressor complexes to silence target genes or activator complexes to activate target gene transcription by regulating histone modifications to activate target gene transcription (124–126). Anna Ehrlund et al. found that SF-1 may regulate target gene expression by adversely influencing Wnt/β-catenin pathways through the inhibition of β-catenin transcription (26), but the impact on oncological phenotypes has not been elucidated completely.

## 6 Epigenetic regulation

### 6.1 EZH2

**Figure 2:**
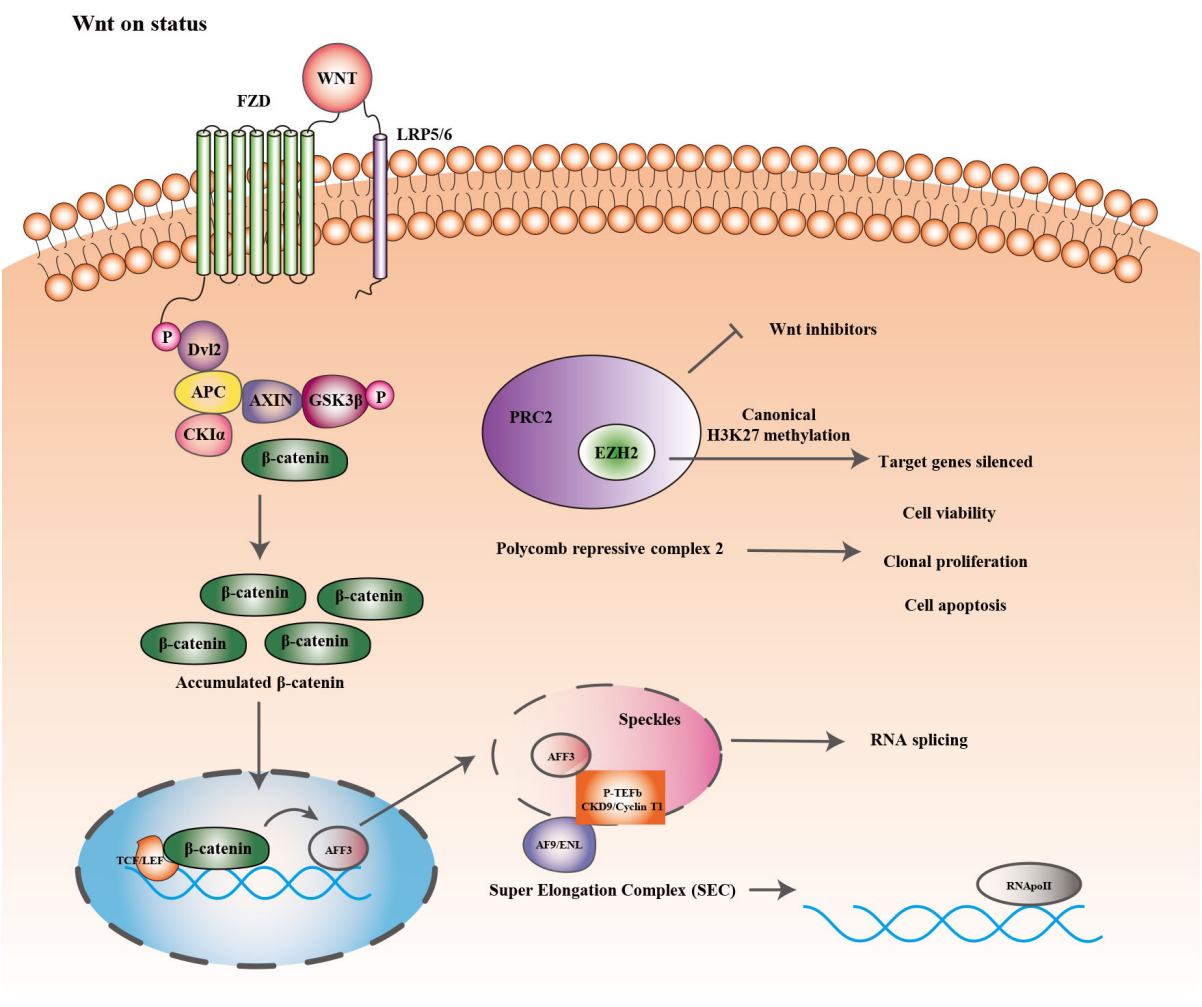
Epigenetic regulation and Wnt/β-catenin signaling pathway.

**Figure 2** showed the epigenetic regulation and Wnt/b-catenin signaling pathway in ACC. Histone methyltransferase (EZH2) is one of the primary catalysis enzymes in the polycomb repressor complex (PRC2) and catalyzes the trimethylation of histone H3 lysine 27 (H3K27me3) to mediate target genes silencing (127). In adrenocortical carcinoma, EZH2 is the most dramatically dysregulated histone modifier, and its overexpression is correlated to poor prognosis and tumor proliferation in patients (27). *In vitro*, RNA interference with EZH2 inhibits H295R cell viability and clonal expansion and induces apoptosis (28). In other tissues, EZH2 stimulates Wnt signaling by inhibiting WNT antagonists (AXIN2, NKD1, PPP2R2B, PRICKLE1, SFRP5, CXXC4) (128–130), but its role in Wnt signaling in ACC has rarely received attention.

### 6.2 AFF3

AFF3 is expressed in the adrenal tissue of mice throughout embryonic development and may be involved in the formation of adrenal glands. According to the immunohistochemical results, B Ragazzon et al. found that AFF3 expression was significantly higher in nuclear β-catenin-stained positive cohorts than in nuclear-stained negative and normal adrenal tissue, while high expression of AFF3 is correlated to poorer OS of patients. Additionally, suppression of either Wnt/β-catenin/TCF or LEF1 decreases AFF3 mRNA levels (29). Regulation of downstream target genes by Wnt/β-catenin generally involves the binding of the transcription factors LEF/TCF to the Wnt response element (WRE), as well as the accumulation of β-catenin. AFF3 has two transcriptional start sites (TSS). In H295R cells, the WRE site, located at nucleotide position -1408 of the AFF3 TSS, participates in regulating Wnt/β-catenin signaling. Earlier studies have discovered that AFF proteins are present in nuclear patches and involved in mRNA shearing (131). The super elongation complex (SEC) composed of the protein AFF (AF4/FMR2), positive transcriptional elongation factor b (P-TEFb), and other elongation factors modulate RNA polymerase II’s transcriptional elongation (132). AFF3 exists in the SEC of adrenocortical cells and its interaction with CDK9 and cell cycle protein T1 (a crucial component of P-TEFb) changes the nuclear distribution of the latter (29). The results above suggest that in adrenocortical cells AFF3 can regulate the activity of P-TEFb. Therefore, the discovery and development of potential antagonists that interfere with the stability or organization of SEC and affect the accumulation of cancer genes to chromatin would be promising anticancer medicines.

### 6.3 RARRES2

Regarded as a secreted ligand of the G protein-coupled receptor chemokine-like receptor 1 (CMKLR1), Retinoic acid receptor response protein 2 (RARRES2) (133, 134) participates in the immunological defense by acting as a chemokine that recruits CMKLR1-expressing immune cells to the positions of injured lymphoid tissues and organs (133). In ACC, CpG hypermethylation silences the expression of RARRES2 with significantly decreased and correlated mRNA and protein expression levels. *In vitro*, overexpression of RARRES2 through transient transfection inhibited cell proliferation and cell invasion but had no significant effect on cell migration. In addition, stable overexpression of RARRES2 resulted in reduced colony formation in clone formation assays and wall-independent cell growth in soft agar colony formation assays. The degree of inhibition was directly proportional to the level of RARRES2 expression, indicating that RARRES2 has a dose-dependent growth inhibitory effect. Mechanistic studies revealed that overexpression promoted β-catenin phosphorylation at Ser33/Ser37/Thr41, leading to increased degradation of β-catenin, thereby reducing total β-catenin levels. Furthermore, the excessive expression of RARRES2 inhibited the activity of the transcription factor TCF/LEF, which in turn inhibited the Wnt/β-catenin signaling pathway and downstream gene expression. Phosphorylated p38 signaling is present in most ACC tumor samples (30). Overexpression of RARRES2 inhibits p38 mitogen-activated protein kinase phosphorylation, which is considered a promising treatment target for adrenocortical tumors (135). Previously conducted studies found that RARRES2 acts as a secreted protein that recruits CMKLR1-expressing NK cells to tumor sites to exert tumor suppressive effects indirectly (133). However, endogenous receptors were not detected in adrenocortical carcinoma cell lines, suggesting that the observed tumor suppressive effects may be independent of immune mechanisms.

## 7 Drugs and inhibitors

From the mechanism aspect, the transmission of the Wnt/β-catenin pathway is regulated at four levels. This also provides new directions for the development of clinical medicine treatment. Including extracellular and cell membrane (expression of WNT ligand, WIF1, DKK), cytoplasm (expression level and stability of β-catenin), cell nucleus (involvement of TCF/LEF, SF1 transcription factors), and crosstalk of other signaling pathways (FGFR, IGF2).

Curcumin, as a natural product derived from turmeric, has been used in the treatment of other cancers (145). Several preclinical studies and clinical applications have reported its therapeutic effect (146, 147). *In vitro*, it has been demonstrated that curcumin has significant antiproliferative effects on a variety of cancer cells through the inhibition of Wnt signaling. EF24, a more soluble curcumin derivative, has similar safety efficiency and higher anticancer activity (148). According to Loris Bertazza’s study, EF24 exerts antiproliferative effects in ACC through multiple pathways, including the Wnt/β-catenin signaling, NF-κB pathway, MAPK pathway, and PI3k/Akt pathway (140).

Nutlin-3a, a classical MDM2 inhibitor, has unexpected pharmacological effects in cancer cells with mutations in CTNNB1. CTNNB1 is a subunit of the calmodulin complex, encodes β-catenin, and acts as an intracellular signaling molecule to activate the Wnt signaling pathway (149). Activating mutations or overexpression of CTNNB1 results in the activation of the Wnt/β-catenin pathway, and is also associated with tumorigenesis in ACC (150). Wen Hui used bioinformatics analysis, and *in vitro* and *in vivo* experiments to demonstrate that Nutlin-3a inhibits several characteristics of H295R cells, including proliferation, EMT, hormone production, and tumorigenesis, making Nutlin-3a an attractive drug for the treatment of CTNNB1-mutated ACC (142).

Rottlerin is a natural plant polyphenol. In recent years, it has been shown anticancer activity in several cancers, such as prostate cancer and pancreatic cancer (151). Yi Zhu demonstrated Rottlerin inhibits cell proliferation, invasion, and metastasis and induces apoptosis and cell cycle arrest by inhibiting Wnt/β-catenin signaling (143).

Abiraterone Acetate is a potent inhibitor of 17alpha-hydroxylase/17,20-lyase (CYP17A1) (152). Due to its inhibition of the synthesis of adrenal androgen, it has been used in metastatic castration-resistant prostate cancer (CRPC). In Sandra Sigala’s view, abiraterone exerts a cytotoxic effect through the progesterone receptor (PgR). In the H295R cell line, abiraterone inhibits nuclear translocation of β-catenin, thereby inhibiting the Wnt/β-catenin signaling (144).

Tegavivint, a newly developed inhibitor of TBL1, is in clinical trials now. On the one hand, it disrupts the binding of β-catenin and the transactivator protein β-like protein 1 (TBL1, a key bridging protein for β-catenin binding and transcriptional activation). On the other hand, it promotes SIAH-1-mediated degradation of nuclear β-catenin (153–156). Tegavivint inhibits the Wnt/β-catenin signaling pathway, decreases the expression of extracellular matrix components, and inhibits cell viability and tumor growth (136). PKF115-584 is a T cell factor/β-catenin antagonist, that dose-dependently inhibits β-catenin-dependent transcription and cell proliferation (17).

Vitamin D receptor is overexpressed in H295R cells due to hypermethylation. By inhibiting Wnt/β-catenin signaling through the activation of VDR, calcitriol inhibits tumor proliferation and growth (137). The combination role of mitotane and calcitriol, regulates the VDR and Wnt/β-catenin signaling, exerting antiproliferative effects (139). Mechanistically, calcitriol, the activated form of vitamin D, promotes β-catenin binding to VDR and reduces binding with the transcription factor TCF/LEF. Current drugs and inhibitors that modulate Wnt/β-catenin are shown in **Table 2**.

**Table 2:** The current drugs and inhibitors.

No.	Name	Target	Intervention mechanism	References
1	Tegavivint	TBL1	Inhibits tumor growth by interfering with β-catenin binding to TBL1	(136)
2	PKF115-584	TCF	Inhibits cell proliferation by interfering with β-catenin binding to TCF	(17)
3	Calcitriol/Seocalcitol	VDR	Suppresses cell proliferation and tumor growth by activating VDR signaling and inhibiting Wnt/β-catenin signaling	(137)
4	Telomelysin	TERT	Inconclusive	(138)
5	Mitotane + 1α,25-dihydroxy vitamin D3	VDR+Wnt/β-catenin	Inhibits cell growth and viability synergistically	(139)
6	EF24	Multiple pathways	Inhibits cell viability, invasion, and clone through multiple pathways	(140)
7	Progesterone	Wnt/β-catenin	Promotes apoptosis	(141)
8	Nutlin-3a	MDM2	Inconclusive	(142)
9	Palbociclib/Ribociclib	CDK4/6	Induces apoptosis, cell cycle arrest, and senescence	(138)
10	Rottlerin	Wnt/β-catenin	Inhibits cell proliferation and invasive metastasis. Induces apoptosis and cell-cycle arrest	(143)
11	Abiraterone	CYP17A1	Inhibits cortisol and androgen, and increases progesterone secretion. Inhibits cell viability and proliferation via PgR	(144)

Telomerase reverse transcriptase (TERT) and regulator of telomere elongation helicase 1 (RTEL1) play key roles in telomere homeostasis (157). Studies have shown that they present in ACC with an increasing number of gene copy and promoter mutations, and relate to clinicopathologic features and poor prognosis. Bioinformatics analysis indicated that high TERT and RTEL1 mRNA levels were associated with the Wnt/β-catenin signaling pathway (158), but there is no experimental proof. Studies of combination therapy of Telomelysin and pembrolizumab for various solid tumors are currently undergoing (NCT03921021, NCT04685499, NCT02293850, NCT03190824). However, whether Telomelysin (OBP-301 and INO5401) can play a role in the treatment of ACC remains to be confirmed.

As an inhibitor of CDK6, Palbociclib has been approved by the FDA and used for the first-line treatment for advanced or metastatic breast cancer with HR^+^ or human epidermal growth factor receptor 2-negative (HER2^-^) (159). Djihad’s vitro experiments demonstrated that palbociclib induced the reduction of active β-catenin, and also inhibited the induced transcription and β-catenin-dependent apoptosis (138).

## 8 Conclusion and prospect

In recent years, there has been significant progress in the studies of targeted drugs and molecular inhibitors as a means to inhibit tumor progression. This has provided a new perspective on the treatment of ACC and opened up exciting possibilities for more effective therapies. Given that ACC pathogenesis has been linked to prolonged stimulation of the Wnt/β-catenin signaling pathway, it is crucial to investigate its involvement as a driving factor. From aspects of Wnt signaling pathway alteration, transcription factor regulation, growth factor signaling pathway, and epigenetic regulation, our review describes the interaction of different molecules and complexes with the Wnt signaling pathway in ACC. At the same time, we summarize the new drugs and inhibitors that regulate the Wnt signaling pathway. In conclusion, obstructing Wnt/β-catenin signaling could be an appropriate alternative treatment for ACC patients and it is crucial to identify methods that can safely and effectively prevent the activation of the β-catenin pathway for patients.

## Author contributions

YT: Writing – original draft. JS: Writing – review & editing.

## References

[1] Else T Kim AC Sabolch A Raymond VM Kandathil A Caoili EM. Adrenocortical carcinoma. Endocr. Rev (2014) 35(2):282–326. doi: 10.1210/er.2013-1029 24423978 PMC3963263

[2] Golden SH Robinson KA Saldanha I Anton B Ladenson PW. Clinical review: Prevalence and incidence of endocrine and metabolic disorders in the United States: a comprehensive review. J Clin Endocrinol Metab (2009) 94(6):1853–78. doi: 10.1210/jc.2008-2291 PMC5393375 19494161

[3] Glenn JA Else T Hughes DT Cohen MS Jolly S Giordano TJ. Longitudinal patterns of recurrence in patients with adrenocortical carcinoma. Surgery (2019) 165(1):186–95. doi: 10.1016/j.surg.2018.04.068 30343951

[4] Baur J Buntemeyer TO Megerle F Deutschbein T Spitzweg C Quinkler M. Outcome after resection of Adrenocortical Carcinoma liver metastases: a retrospective study. BMC Cancer (2017) 17(1):522. doi: 10.1186/s12885-017-3506-z 28778197 PMC5545028

[5] Fassnacht M Kroiss M Allolio B. Update in adrenocortical carcinoma. J Clin Endocrinol Metab (2013) 98(12):4551–64. doi: 10.1210/jc.2013-3020 24081734

[6] Postlewait LM Ethun CG Tran TB Prescott JD Pawlik TM Wang TS. Outcomes of adjuvant mitotane after resection of adrenocortical carcinoma: A 13-institution study by the US adrenocortical carcinoma group. J Am Coll Surg (2016) 222(4):480–90. doi: 10.1016/j.jamcollsurg.2015.12.013 PMC4957938 26775162

[7] Kroiss M Quinkler M Lutz WK Allolio B Fassnacht M. Drug interactions with mitotane by induction of CYP3A4 metabolism in the clinical management of adrenocortical carcinoma. Clin Endocrinol (Oxf) (2011) 75(5):585–91. doi: 10.1111/j.1365-2265.2011.04214.x 21883349

[8] Mir MC Klink JC Guillotreau J Long JA Miocinovic R Kaouk JH. Comparative outcomes of laparoscopic and open adrenalectomy for adrenocortical carcinoma: single, high-volume center experience. Ann Surg Oncol (2013) 20(5):1456–61. doi: 10.1245/s10434-012-2760-1 23184291

[9] Kuulasmaa T Jaaskelainen J Suppola S Pietilainen T Heikkila P Aaltomaa S. WNT-4 mRNA expression in human adrenocortical tumors and cultured adrenal cells. Horm. Metab Res (2008) 40(10):668–73. doi: 10.1055/s-2008-1078716 18553255

[10] Feng Y Wang Y Guo K Feng J Shao C Pan M. The value of WNT5A as prognostic and immunological biomarker in pan-cancer. Ann Transl Med (2022) 10(8):466. doi: 10.21037/atm-22-1317 35571400 PMC9096401

[11] Mitsui Y Yasumoto H Nagami T Hiraki M Arichi N Ishikawa N. Extracellular activation of Wnt signaling through epigenetic dysregulation of Wnt inhibitory factor-1 (Wif-1) is associated with pathogenesis of adrenocortical tumor. Oncotarget (2014) 5(8):2198–207. doi: 10.18632/oncotarget.1889 PMC4039156 24755523

[12] Cheng JY Brown TC Murtha TD Stenman A Juhlin CC Larsson C. A novel FOXO1-mediated dedifferentiation blocking role for DKK3 in adrenocortical carcinogenesis. BMC Cancer (2017) 17(1):164. doi: 10.1186/s12885-017-3152-5 28249601 PMC5333434

[13] Korah R Healy JM Kunstman JW Fonseca AL Ameri AH Prasad ML. Epigenetic silencing of RASSF1A deregulates cytoskeleton and promotes Malignant behavior of adrenocortical carcinoma. Mol Cancer (2013) 12:87. doi: 10.1186/1476-4598-12-87 23915220 PMC3750604

[14] Durand J Lampron A Mazzuco TL Chapman A Bourdeau I. Characterization of differential gene expression in adrenocortical tumors harboring beta-catenin (CTNNB1) mutations. J Clin Endocrinol Metab (2011) 96(7):E1206–11. doi: 10.1210/jc.2010-2143 21565795

[15] Leal LF Bueno AC Gomes DC Abduch R de Castro M Antonini SR. Inhibition of the Tcf/beta-catenin complex increases apoptosis and impairs adrenocortical tumor cell proliferation and adrenal steroidogenesis. Oncotarget (2015) 6(40):43016–32. doi: 10.18632/oncotarget.5513 PMC4767488 26515592

[16] Gaujoux S Hantel C Launay P Bonnet S Perlemoine K Lefevre L. Silencing mutated beta-catenin inhibits cell proliferation and stimulates apoptosis in the adrenocortical cancer cell line H295R. PloS One (2013) 8(2):e55743. doi: 10.1371/journal.pone.0055743 23409032 PMC3567123

[17] Doghman M Cazareth J Lalli E. The T cell factor/beta-catenin antagonist PKF115-584 inhibits proliferation of adrenocortical carcinoma cells. J Clin Endocrinol Metab (2008) 93(8):3222–5. doi: 10.1210/jc.2008-0247 18544621

[18] Brown TC Nicolson NG Korah R Carling T. BCL9 upregulation in adrenocortical carcinoma: A novel Wnt/beta-catenin activating event driving adrenocortical Malignancy. J Am Coll Surg (2018) 226(6):988–95. doi: 10.1016/j.jamcollsurg.2018.01.051 29428231

[19] Abduch RH Carolina BA Leal LF Cavalcanti MM Gomes DC Brandalise SR. Unraveling the expression of the oncogene YAP1, a Wnt/beta-catenin target, in adrenocortical tumors and its association with poor outcome in pediatric patients. Oncotarget (2016) 7(51):84634–44. doi: 10.18632/oncotarget.12382 PMC5356687 27705928

[20] Maria AG Silva BK Lira R Hassib TC Berthon A Drougat L. Inhibition of Aurora kinase A activity enhances the antitumor response of beta-catenin blockade in human adrenocortical cancer cells. Mol Cell Endocrinol (2021) 528:111243. doi: 10.1016/j.mce.2021.111243 33716050 PMC8297658

[21] Borges KS Andrade AF Silveira VS Marco AD Vasconcelos E Antonini S. The aurora kinase inhibitor AMG 900 increases apoptosis and induces chemosensitivity to anticancer drugs in the NCI-H295 adrenocortical carcinoma cell line. Anticancer Drugs (2017) 28(6):634–44. doi: 10.1097/CAD.0000000000000504 28410270

[22] He H Dai J Yang X Wang X Sun F Zhu Y. Silencing of MED27 inhibits adrenal cortical carcinogenesis by targeting the Wnt/beta-catenin signaling pathway and the epithelial-mesenchymal transition process. Biol Chem (2018) 399(6):593–602. doi: 10.1515/hsz-2017-0304 29730647

[23] Haase M Schott M Bornstein SR Malendowicz LK Scherbaum WA Willenberg HS. CITED2 is expressed in human adrenocortical cells and regulated by basic fibroblast growth factor. J Endocrinol (2007) 192(2):459–65. doi: 10.1677/JOE-06-0083 17283246

[24] Krejci P Aklian A Kaucka M Sevcikova E Prochazkova J Masek JK. Receptor tyrosine kinases activate canonical WNT/beta-catenin signaling via MAP kinase/LRP6 pathway and direct beta-catenin phosphorylation. PloS One (2012) 7(4):e35826. doi: 10.1371/journal.pone.0035826 22558232 PMC3338780

[25] Heaton JH Wood MA Kim AC Lima LO Barlaskar FM Almeida MQ. Progression to adrenocortical tumorigenesis in mice and humans through insulin-like growth factor 2 and beta-catenin. Am J Pathol (2012) 181(3):1017–33. doi: 10.1016/j.ajpath.2012.05.026 PMC3432433 22800756

[26] Ehrlund A Jonsson P Vedin LL Williams C Gustafsson JA Treuter E. Knockdown of SF-1 and RNF31 affects components of steroidogenesis, TGFbeta, and Wnt/beta-catenin signaling in adrenocortical carcinoma cells. PloS One (2012) 7(3):e32080. doi: 10.1371/journal.pone.0032080 22427816 PMC3302881

[27] Xing Z Luo Z Yang H Huang Z Liang X. Screening and identification of key biomarkers in adrenocortical carcinoma based on bioinformatics analysis. Oncol Lett (2019) 18(5):4667–76. doi: 10.3892/ol.2019.10817 PMC6781718 31611976

[28] Drelon C Berthon A Mathieu M Ragazzon B Kuick R Tabbal H. Val: EZH2 is overexpressed in adrenocortical carcinoma and is associated with disease progression. Hum Mol Genet (2016) 25(13):2789–800. doi: 10.1093/hmg/ddw136 PMC6095221 27149985

[29] Lefevre L Omeiri H Drougat L Hantel C Giraud M Val P. Combined transcriptome studies identify AFF3 as a mediator of the oncogenic effects of beta-catenin in adrenocortical carcinoma. Oncogenesis (2015) 4(7):e161. doi: 10.1038/oncsis.2015.20 26214578 PMC4521181

[30] Liu-Chittenden Y Jain M Gaskins K Wang S Merino MJ Kotian S. RARRES2 functions as a tumor suppressor by promoting beta-catenin phosphorylation/degradation and inhibiting p38 phosphorylation in adrenocortical carcinoma. Oncogene (2017) 36(25):3541–52. doi: 10.1038/onc.2016.497 PMC5481486 28114280

[31] Bryja V Cervenka I Cajanek L. The connections of Wnt pathway components with cell cycle and centrosome: side effects or a hidden logic? Crit Rev Biochem Mol Biol (2017) 52(6):614–37. doi: 10.1080/10409238.2017.1350135 PMC6047740 28741966

[32] Zhang Y Wang X. Targeting the Wnt/beta-catenin signaling pathway in cancer. J Hematol Oncol (2020) 13(1):165. doi: 10.1186/s13045-020-00990-3 33276800 PMC7716495

[33] Neiheisel A Kaur M Ma N Havard P Shenoy AK. Wnt pathway modulators in cancer therapeutics: An update on completed and ongoing clinical trials. Int J Cancer (2022) 150(5):727–40. doi: 10.1002/ijc.33811 34536299

[34] Miete C Solis GP Koval A Bruckner M Katanaev VL Behrens J. Galphai2-induced conductin/axin2 condensates inhibit Wnt/beta-catenin signaling and suppress cancer growth. Nat Commun (2022) 13(1):674. doi: 10.1038/s41467-022-28286-9 35115535 PMC8814139

[35] Parichha A Suresh V Chatterjee M Kshirsagar A Ben-Reuven L Olender T. Constitutive activation of canonical Wnt signaling disrupts choroid plexus epithelial fate. Nat Commun (2022) 13(1):633. doi: 10.1038/s41467-021-27602-z 35110543 PMC8810795

[36] Nusse R Clevers H. Wnt/beta-catenin signaling, disease, and emerging therapeutic modalities. Cell (2017) 169(6):985–99. doi: 10.1016/j.cell.2017.05.016 28575679

[37] Shi J Li F Luo M Wei J Liu X. Distinct roles of Wnt/beta-catenin signaling in the pathogenesis of chronic obstructive pulmonary disease and idiopathic pulmonary fibrosis. Mediators Inflammation (2017) 2017:3520581. doi: 10.1155/2017/3520581 PMC5447271 28588349

[38] Minde DP Anvarian Z Rudiger SG Maurice MM. Messing up disorder: how do missense mutations in the tumor suppressor protein APC lead to cancer? Mol Cancer (2011) 10:101. doi: 10.1186/1476-4598-10-101 21859464 PMC3170638

[39] Zeng L Fagotto F Zhang T Hsu W Vasicek TJ Perry WR. The mouse Fused locus encodes Axin, an inhibitor of the Wnt signaling pathway that regulates embryonic axis formation. Cell (1997) 90(1):181–92. doi: 10.1016/s0092-8674(00)80324-4 9230313

[40] Cruciat CM. Casein kinase 1 and Wnt/beta-catenin signaling. Curr Opin Cell Biol (2014) 31:46–55. doi: 10.1016/j.ceb.2014.08.003 25200911

[41] Wu D Pan W. GSK3: a multifaceted kinase in Wnt signaling. Trends Biochem Sci (2010) 35(3):161–8. doi: 10.1016/j.tibs.2009.10.002 PMC2834833 19884009

[42] MacDonald BT Tamai K He X. Wnt/beta-catenin signaling: components, mechanisms, and diseases. Dev Cell (2009) 17(1):9–26. doi: 10.1016/j.devcel.2009.06.016 19619488 PMC2861485

[43] Liu C Li Y Semenov M Han C Baeg GH Tan Y. Control of beta-catenin phosphorylation/degradation by a dual-kinase mechanism. Cell (2002) 108(6):837–47. doi: 10.1016/s0092-8674(02)00685-2 11955436

[44] Rao TP Kuhl M. An updated overview on Wnt signaling pathways: a prelude for more. Circ Res (2010) 106(12):1798–806. doi: 10.1161/CIRCRESAHA.110.219840 20576942

[45] Tewari D Bawari S Sharma S DeLiberto LK Bishayee A. Targeting the crosstalk between canonical Wnt/beta-catenin and inflammatory signaling cascades: A novel strategy for cancer prevention and therapy. Pharmacol Ther (2021) 227:107876. doi: 10.1016/j.pharmthera.2021.107876 33930452

[46] Kwon C Cheng P King IN Andersen P Shenje L Nigam V. Notch post-translationally regulates beta-catenin protein in stem and progenitor cells. Nat Cell Biol (2011) 13(10):1244–51. doi: 10.1038/ncb2313 PMC3187850 21841793

[47] Moroney MR Woodruff E Qamar L Bradford AP Wolsky R Bitler BG. Inhibiting Wnt/beta-catenin in CTNNB1-mutated endometrial cancer. Mol Carcinog (2021) 60(8):511–23. doi: 10.1002/mc.23308 34038589

[48] Cieply B Zeng G Proverbs-Singh T Geller DA Monga SP. Unique phenotype of hepatocellular cancers with exon-3 mutations in beta-catenin gene. Hepatology (2009) 49(3):821–31. doi: 10.1002/hep.22695 PMC2657345 19101982

[49] Gaujoux S Grabar S Fassnacht M Ragazzon B Launay P Libe R. beta-catenin activation is associated with specific clinical and pathologic characteristics and a poor outcome in adrenocortical carcinoma. Clin Cancer Res (2011) 17(2):328–36. doi: 10.1158/1078-0432.CCR-10-2006 21088256

[50] Jung YS Park JI. Wnt signaling in cancer: therapeutic targeting of Wnt signaling beyond beta-catenin and the destruction complex. Exp Mol Med (2020) 52(2):183–91. doi: 10.1038/s12276-020-0380-6 PMC7062731 32037398

[51] van Amerongen R. Alternative Wnt pathways and receptors. Cold Spring Harb Perspect Biol (2012) 4(10):715–22. doi: 10.1101/cshperspect.a007914 PMC3475174 22935904

[52] VanderVorst K Dreyer CA Konopelski SE Lee H Ho HH Carraway KR. Wnt/PCP signaling contribution to carcinoma collective cell migration and metastasis. Cancer Res (2019) 79(8):1719–29. doi: 10.1158/0008-5472.CAN-18-2757 PMC6467734 30952630

[53] De A. Wnt/Ca2+ signaling pathway: a brief overview. Acta Biochim Biophys Sin (Shanghai) (2011) 43(10):745–56. doi: 10.1093/abbs/gmr079 21903638

[54] Park HW Kim YC Yu B Moroishi T Mo JS Plouffe SW. Alternative Wnt signaling activates YAP/TAZ. Cell (2015) 162(4):780–94. doi: 10.1016/j.cell.2015.07.013 PMC4538707 26276632

[55] Gujral TS Chan M Peshkin L Sorger PK Kirschner MW MacBeath G. A noncanonical Frizzled2 pathway regulates epithelial-mesenchymal transition and metastasis. Cell (2014) 159(4):844–56. doi: 10.1016/j.cell.2014.10.032 PMC4243058 25417160

[56] Green J Nusse R van Amerongen R. The role of Ryk and Ror receptor tyrosine kinases in Wnt signal transduction. Cold Spring Harb Perspect Biol (2014) 6(2):482–90. doi: 10.1101/cshperspect.a009175 PMC3941236 24370848

[57] Parsons MJ Tammela T Dow LE. WNT as a driver and dependency in cancer. Cancer Discovery (2021) 11(10):2413–29. doi: 10.1158/2159-8290.CD-21-0190 PMC8487948 34518209

[58] Zeng G Awan F Otruba W Muller P Apte U Tan X. Wnt’er in liver: expression of Wnt and frizzled genes in mouse. Hepatology (2007) 45(1):195–204. doi: 10.1002/hep.21473 17187422

[59] Urakami S Shiina H Enokida H Kawakami T Tokizane T Ogishima T. Epigenetic inactivation of Wnt inhibitory factor-1 plays an important role in bladder cancer through aberrant canonical Wnt/beta-catenin signaling pathway. Clin Cancer Res (2006) 12(2):383–91. doi: 10.1158/1078-0432.CCR-05-1344 16428476

[60] Kawakami K Hirata H Yamamura S Kikuno N Saini S Majid S. Functional significance of Wnt inhibitory factor-1 gene in kidney cancer. Cancer Res (2009) 69(22):8603–10. doi: 10.1158/0008-5472.CAN-09-2534 19887605

[61] Veeck J Dahl E. Targeting the Wnt pathway in cancer: the emerging role of Dickkopf-3. Biochim Biophys Acta (2012) 1825(1):18–28. doi: 10.1016/j.bbcan.2011.09.003 21982838

[62] Lee EJ Jo M Rho SB Park K Yoo YN Park J. Dkk3, downregulated in cervical cancer, functions as a negative regulator of beta-catenin. Int J Cancer (2009) 124(2):287–97. doi: 10.1002/ijc.23913 19003969

[63] Yin DT Wu W Li M Wang QE Li H Wang Y. DKK3 is a potential tumor suppressor gene in papillary thyroid carcinoma. Endocr Relat Cancer (2013) 20(4):507–14. doi: 10.1530/ERC-13-0053 23702469

[64] Hsieh SY Hsieh PS Chiu CT Chen WY. Dickkopf-3/REIC functions as a suppressor gene of tumor growth. Oncogene (2004) 23(57):9183–9. doi: 10.1038/sj.onc.1208138 15516983

[65] Veeck J Bektas N Hartmann A Kristiansen G Heindrichs U Knuchel R. Wnt signalling in human breast cancer: expression of the putative Wnt inhibitor Dickkopf-3 (DKK3) is frequently suppressed by promoter hypermethylation in mammary tumours. Breast Cancer Res (2008) 10(5):R82. doi: 10.1186/bcr2151 18826564 PMC2614517

[66] Liang L He H Lv R Zhang M Huang H An Z. Preliminary mechanism on the methylation modification of Dkk-1 and Dkk-3 in hepatocellular carcinoma. Tumour Biol (2015) 36(2):1245–50. doi: 10.1007/s13277-014-2750-y 25344678

[67] Hoang BH Kubo T Healey JH Yang R Nathan SS Kolb EA. Dickkopf 3 inhibits invasion and motility of Saos-2 osteosarcoma cells by modulating the Wnt-beta-catenin pathway. Cancer Res (2004) 64(8):2734–9. doi: 10.1158/0008-5472.can-03-1952 15087387

[68] Saeb-Parsy K Veerakumarasivam A Wallard MJ Thorne N Kawano Y Murphy G. MT1-MMP regulates urothelial cell invasion via transcriptional regulation of Dickkopf-3. Br J Cancer (2008) 99(4):663–9. doi: 10.1038/sj.bjc.6604513 PMC2527828 18665176

[69] El WA Bandulik S Guy N Bendahhou S Zennaro MC Niehrs C. Dkk3 is a component of the genetic circuitry regulating aldosterone biosynthesis in the adrenal cortex. Hum Mol Genet (2012) 21(22):4922–9. doi: 10.1093/hmg/dds333 22918120

[70] Zheng S Cherniack AD Dewal N Moffitt RA Danilova L Murray BA. Comprehensive pan-genomic characterization of adrenocortical carcinoma. Cancer Cell (2016) 29(5):723–36. doi: 10.1016/j.ccell.2016.04.002 PMC4864952 27165744

[71] Juhlin CC Goh G Healy JM Fonseca AL Scholl UI Stenman A. Whole-exome sequencing characterizes the landscape of somatic mutations and copy number alterations in adrenocortical carcinoma. J Clin Endocrinol Metab (2015) 100(3):E493–502. doi: 10.1210/jc.2014-3282 PMC5393505 25490274

[72] Petrie RJ Yamada KM. At the leading edge of three-dimensional cell migration. J Cell Sci (2012) 125(Pt 24):5917–26. doi: 10.1242/jcs.093732 PMC4067260 23378019

[73] Krause M Gautreau A. Steering cell migration: lamellipodium dynamics and the regulation of directional persistence. Nat Rev Mol Cell Biol (2014) 15(9):577–90. doi: 10.1038/nrm3861 25145849

[74] Petrie RJ Gavara N Chadwick RS Yamada KM. Nonpolarized signaling reveals two distinct modes of 3D cell migration. J Cell Biol (2012) 197(3):439–55. doi: 10.1083/jcb.201201124 PMC3341168 22547408

[75] Stenman A Murtha T Korah R Carling T. Suppression of forkhead box protein O1 (FOXO1) transcription factor may promote adrenocortical tumorigenesis. Horm. Metab Res (2017) 49(8):631–7. doi: 10.1055/s-0043-110143 28641336

[76] Kamilaris C Hannah-Shmouni F Stratakis CA. Adrenocortical tumorigenesis: Lessons from genetics. Best Pract Res Clin Endocrinol Metab (2020) 34(3):101428. doi: 10.1016/j.beem.2020.101428 32507359 PMC7427505

[77] Zhou T Luo P Wang L Yang S Qin S Wei Z. CTNNB1 knockdown inhibits cell proliferation and aldosterone secretion through inhibiting Wnt/beta-catenin signaling in H295R cells. Technol Cancer Res Treat (2020) 19:1533033820979685. doi: 10.1177/1533033820979685 33287648 PMC7727057

[78] Borges KS Pignatti E Leng S Kariyawasam D Ruiz-Babot G Ramalho FS. Wnt/beta-catenin activation cooperates with loss of p53 to cause adrenocortical carcinoma in mice. Oncogene (2020) 39(30):5282–91. doi: 10.1038/s41388-020-1358-5 PMC7378041 32561853

[79] Sampietro J Dahlberg CL Cho US Hinds TR Kimelman D Xu W. Crystal structure of a beta-catenin/BCL9/Tcf4 complex. Mol Cell (2006) 24(2):293–300. doi: 10.1016/j.molcel.2006.09.001 17052462

[80] Wang Z Li Z Ji H. Direct targeting of beta-catenin in the Wnt signaling pathway: Current progress and perspectives. Med Res Rev (2021) 41(4):2109–29. doi: 10.1002/med.21787 PMC8217106 33475177

[81] Takada K Zhu D Bird GH Sukhdeo K Zhao JJ Mani M. Targeted disruption of the BCL9/beta-catenin complex inhibits oncogenic Wnt signaling. Sci Transl Med (2012) 4(148):148ra117. doi: 10.1126/scitranslmed.3003808 PMC3631420 22914623

[82] Xu W Zhou W Cheng M Wang J Liu Z He S. Hypoxia activates Wnt/beta-catenin signaling by regulating the expression of BCL9 in human hepatocellular carcinoma. Sci Rep (2017) 7:40446. doi: 10.1038/srep40446 28074862 PMC5225427

[83] Wang J Ying Y Bo S Li G Yuan F. Differentially expressed microRNA-218 modulates the viability of renal cell carcinoma by regulating BCL9. Mol Med Rep (2016) 14(2):1829–34. doi: 10.3892/mmr.2016.5403 27314976

[84] Elsarraj HS Hong Y Valdez KE Michaels W Hook M Smith WP. Expression profiling of in *vivo* ductal carcinoma in *situ* progression models identified B cell lymphoma-9 as a molecular driver of breast cancer invasion. Breast Cancer Res (2015) 17:128. doi: 10.1186/s13058-015-0630-z 26384318 PMC4574212

[85] Mani M Carrasco DE Zhang Y Takada K Gatt ME Dutta-Simmons J. BCL9 promotes tumor progression by conferring enhanced proliferative, metastatic, and angiogenic properties to cancer cells. Cancer Res (2009) 69(19):7577–86. doi: 10.1158/0008-5472.CAN-09-0773 PMC4321734 19738061

[86] Moor AE Anderle P Cantu C Rodriguez P Wiedemann N Baruthio F. BCL9/9L-beta-catenin signaling is associated with poor outcome in colorectal cancer. EBioMedicine (2015) 2(12):1932–43. doi: 10.1016/j.ebiom.2015.10.030 PMC4703711 26844272

[87] Patel S Alam A Pant R Chattopadhyay S. Wnt signaling and its significance within the tumor microenvironment: novel therapeutic insights. Front Immunol (2019) 10:2872. doi: 10.3389/fimmu.2019.02872 31921137 PMC6927425

[88] Jiang M Kang Y Sewastianik T Wang J Tanton H Alder K. BCL9 provides multi-cellular communication properties in colorectal cancer by interacting with paraspeckle proteins. Nat Commun (2020) 11(1):19. doi: 10.1038/s41467-019-13842-7 31911584 PMC6946813

[89] Zhang H Bao Y Liu C Li J Zhu D Zhang Q. Recent advances in beta-catenin/BCL9 protein-protein interaction inhibitors. Future Med Chem (2021) 13(10):927–40. doi: 10.4155/fmc-2020-0357 33849283

[90] Avruch J Zhou D Bardeesy N. YAP oncogene overexpression supercharges colon cancer proliferation. Cell Cycle (2012) 11(6):1090–6. doi: 10.4161/cc.11.6.19453 PMC3335916 22356765

[91] Camargo FD Gokhale S Johnnidis JB Fu D Bell GW Jaenisch R. YAP1 increases organ size and expands undifferentiated progenitor cells. Curr Biol (2007) 17(23):2054–60. doi: 10.1016/j.cub.2007.10.039 17980593

[92] Overholtzer M Zhang J Smolen GA Muir B Li W Sgroi DC. Transforming properties of YAP, a candidate oncogene on the chromosome 11q22 amplicon. Proc Natl Acad Sci U.S.A. (2006) 103(33):12405–10. doi: 10.1073/pnas.0605579103 PMC1533802 16894141

[93] Fernandez-L A Northcott PA Dalton J Fraga C Ellison D Angers S. YAP1 is amplified and up-regulated in hedgehog-associated medulloblastomas and mediates Sonic hedgehog-driven neural precursor proliferation. Genes Dev (2009) 23(23):2729–41. doi: 10.1101/gad.1824509 PMC2788333 19952108

[94] Konsavage WJ Yochum GS. Intersection of Hippo/YAP and Wnt/beta-catenin signaling pathways. Acta Biochim Biophys Sin (Shanghai) (2013) 45(2):71–9. doi: 10.1093/abbs/gms084 23027379

[95] Barry ER Morikawa T Butler BL Shrestha K de la Rosa R Yan KS. Restriction of intestinal stem cell expansion and the regenerative response by YAP. Nature (2013) 493(7430):106–10. doi: 10.1038/nature11693 PMC3536889 23178811

[96] Konsavage WJ Kyler SL Rennoll SA Jin G Yochum GS. Wnt/beta-catenin signaling regulates Yes-associated protein (YAP) gene expression in colorectal carcinoma cells. J Biol Chem (2012) 287(15):11730–9. doi: 10.1074/jbc.M111.327767 PMC3320921 22337891

[97] Wang S Zhou L Ling L Meng X Chu F Zhang S. The crosstalk between hippo-YAP pathway and innate immunity. Front Immunol (2020) 11:323. doi: 10.3389/fimmu.2020.00323 32174922 PMC7056731

[98] Dasgupta I McCollum D. Control of cellular responses to mechanical cues through YAP/TAZ regulation. J Biol Chem (2019) 294(46):17693–706. doi: 10.1074/jbc.REV119.007963 PMC6873206 31594864

[99] Schlegelmilch K Mohseni M Kirak O Pruszak J Rodriguez JR Zhou D. Yap1 acts downstream of alpha-catenin to control epidermal proliferation. Cell (2011) 144(5):782–95. doi: 10.1016/j.cell.2011.02.031 PMC3237196 21376238

[100] Wei SC Fattet L Tsai JH Guo Y Pai VH Majeski HE. Matrix stiffness drives epithelial-mesenchymal transition and tumour metastasis through a TWIST1-G3BP2 mechanotransduction pathway. Nat Cell Biol (2015) 17(5):678–88. doi: 10.1038/ncb3157 PMC4452027 25893917

[101] Willems E Dedobbeleer M Digregorio M Lombard A Lumapat PN Rogister B. The functional diversity of Aurora kinases: a comprehensive review. Cell Div (2018) 13:7. doi: 10.1186/s13008-018-0040-6 30250494 PMC6146527

[102] Tayyar Y Jubair L Fallaha S McMillan N. Critical risk-benefit assessment of the novel anti-cancer aurora a kinase inhibitor alisertib (MLN8237): A comprehensive review of the clinical data. Crit Rev Oncol Hematol (2017) 119:59–65. doi: 10.1016/j.critrevonc.2017.09.006 29065986

[103] Allen BL Taatjes DJ. The Mediator complex: a central integrator of transcription. Nat Rev Mol Cell Biol (2015) 16(3):155–66. doi: 10.1038/nrm3951 PMC4963239 25693131

[104] Casamassimi A Napoli C. Mediator complexes and eukaryotic transcription regulation: an overview. Biochimie (2007) 89(12):1439–46. doi: 10.1016/j.biochi.2007.08.002 17870225

[105] Conaway RC Sato S Tomomori-Sato C Yao T Conaway JW. The mammalian Mediator complex and its role in transcriptional regulation. Trends Biochem Sci (2005) 30(5):250–5. doi: 10.1016/j.tibs.2005.03.002 15896743

[106] Soutourina J. Transcription regulation by the Mediator complex. Nat Rev Mol Cell Biol (2018) 19(4):262–74. doi: 10.1038/nrm.2017.115 29209056

[107] Basile DP Holzwarth MA. Basic fibroblast growth factor may mediate proliferation in the compensatory adrenal growth response. Am J Physiol (1993) 265(6 Pt 2):R1253–61. doi: 10.1152/ajpregu.1993.265.6.R1253 8285265

[108] Boulle N Gicquel C Logie A Christol R Feige JJ Le Bouc Y. Fibroblast growth factor-2 inhibits the maturation of pro-insulin-like growth factor-II (Pro-IGF-II) and the expression of insulin-like growth factor binding protein-2 (IGFBP-2) in the human adrenocortical tumor cell line NCI-H295R. Endocrinology (2000) 141(9):3127–36. doi: 10.1210/endo.141.9.7632 10965883

[109] Feige JJ Vilgrain I Brand C Bailly S Souchelnitskiy S. Fine tuning of adrenocortical functions by locally produced growth factors. J Endocrinol (1998) 158(1):7–19. doi: 10.1677/joe.0.1580007 9713321

[110] Dienstmann R Rodon J Prat A Perez-Garcia J Adamo B Felip E. Genomic aberrations in the FGFR pathway: opportunities for targeted therapies in solid tumors. Ann Oncol (2014) 25(3):552–63. doi: 10.1093/annonc/mdt419 PMC4433501 24265351

[111] Katoh Y Katoh M. FGFR2-related pathogenesis and FGFR2-targeted therapeutics (Review). Int J Mol Med (2009) 23(3):307–11. doi: 10.3892/ijmm_00000132 19212647

[112] Guasti L Candy SW McKay T Grose R King PJ. FGF signalling through Fgfr2 isoform IIIb regulates adrenal cortex development. Mol Cell Endocrinol (2013) 371(1-2):182–8. doi: 10.1016/j.mce.2013.01.014 PMC3650577 23376610

[113] Hafner R Bohnenpoll T Rudat C Schultheiss TM Kispert A. Fgfr2 is required for the expansion of the early adrenocortical primordium. Mol Cell Endocrinol (2015) 413:168–77. doi: 10.1016/j.mce.2015.06.022 26141512

[114] Chae YK Ranganath K Hammerman PS Vaklavas C Mohindra N Kalyan A. Inhibition of the fibroblast growth factor receptor (FGFR) pathway: the current landscape and barriers to clinical application. Oncotarget (2017) 8(9):16052–74. doi: 10.18632/oncotarget.14109 PMC5362545 28030802

[115] Maharjan R Backman S Akerstrom T Hellman P Bjorklund P. Comprehensive analysis of CTNNB1 in adrenocortical carcinomas: Identification of novel mutations and correlation to survival. Sci Rep (2018) 8(1):8610. doi: 10.1038/s41598-018-26799-2 29872083 PMC5988720

[116] Haase M Thiel A Scholl UI Ashmawy H Schott M Ehlers M. Subcellular localization of fibroblast growth factor receptor type 2 and correlation with CTNNB1 genotype in adrenocortical carcinoma. BMC Res Notes (2020) 13(1):282. doi: 10.1186/s13104-020-05110-5 32522271 PMC7288682

[117] Ranieri D Rosato B Nanni M Magenta A Belleudi F Torrisi MR. Expression of the FGFR2 mesenchymal splicing variant in epithelial cells drives epithelial-mesenchymal transition. Oncotarget (2016) 7(5):5440–60. doi: 10.18632/oncotarget.6706 PMC4868697 26713601

[118] Brouwer-Visser J Huang GS. IGF2 signaling and regulation in cancer. Cytokine Growth Factor Rev (2015) 26(3):371–7. doi: 10.1016/j.cytogfr.2015.01.002 25704323

[119] Drelon C Berthon A Ragazzon B Tissier F Bandiera R Sahut-Barnola I. Analysis of the role of Igf2 in adrenal tumour development in transgenic mouse models. PloS One (2012) 7(8):e44171. doi: 10.1371/journal.pone.0044171 22952916 PMC3429465

[120] Guillaud-Bataille M Ragazzon B de Reynies A Chevalier C Francillard I Barreau O. IGF2 promotes growth of adrenocortical carcinoma cells, but its overexpression does not modify phenotypic and molecular features of adrenocortical carcinoma. PloS One (2014) 9(8):e103744. doi: 10.1371/journal.pone.0103744 25089899 PMC4121173

[121] Schimmer BP White PC. Minireview: steroidogenic factor 1: its roles in differentiation, development, and disease. Mol Endocrinol (2010) 24(7):1322–37. doi: 10.1210/me.2009-0519 PMC5417463 20203099

[122] Luo X Ikeda Y Parker KL. A cell-specific nuclear receptor is essential for adrenal and gonadal development and sexual differentiation. Cell (1994) 77(4):481–90. doi: 10.1016/0092-8674(94)90211-9 8187173

[123] Sadovsky Y Crawford PA Woodson KG Polish JA Clements MA Tourtellotte LM. Mice deficient in the orphan receptor steroidogenic factor 1 lack adrenal glands and gonads but express P450 side-chain-cleavage enzyme in the placenta and have normal embryonic serum levels of corticosteroids. Proc Natl Acad Sci U.S.A. (1995) 92(24):10939–43. doi: 10.1073/pnas.92.24.10939 PMC40546 7479914

[124] Ehrlund A Anthonisen EH Gustafsson N Venteclef N Robertson RK Damdimopoulos AE. E3 ubiquitin ligase RNF31 cooperates with DAX-1 in transcriptional repression of steroidogenesis. Mol Cell Biol (2009) 29(8):2230–42. doi: 10.1128/MCB.00743-08 PMC2663311 19237537

[125] Dammer EB Leon A Sewer MB. Coregulator exchange and sphingosine-sensitive cooperativity of steroidogenic factor-1, general control nonderepressed 5, p54, and p160 coactivators regulate cyclic adenosine 3’,5’-monophosphate-dependent cytochrome P450c17 transcription rate. Mol Endocrinol (2007) 21(2):415–38. doi: 10.1210/me.2006-0361 17121866

[126] Winnay JN Hammer GD. Adrenocorticotropic hormone-mediated signaling cascades coordinate a cyclic pattern of steroidogenic factor 1-dependent transcriptional activation. Mol Endocrinol (2006) 20(1):147–66. doi: 10.1210/me.2005-0215 16109736

[127] Cao R Zhang Y. The functions of E(Z)/EZH2-mediated methylation of lysine 27 in histone H3. Curr Opin Genet Dev (2004) 14(2):155–64. doi: 10.1016/j.gde.2004.02.001 15196462

[128] Jung HY Jun S Lee M Kim HC Wang X Ji H. PAF and EZH2 induce Wnt/beta-catenin signaling hyperactivation. Mol Cell (2013) 52(2):193–205. doi: 10.1016/j.molcel.2013.08.028 24055345 PMC4040269

[129] Cheng AS Lau SS Chen Y Kondo Y Li MS Feng H. EZH2-mediated concordant repression of Wnt antagonists promotes beta-catenin-dependent hepatocarcinogenesis. Cancer Res (2011) 71(11):4028–39. doi: 10.1158/0008-5472.CAN-10-3342 21512140

[130] Lu H Sun J Wang F Feng L Ma Y Shen Q. Enhancer of zeste homolog 2 activates wnt signaling through downregulating CXXC finger protein 4. Cell Death Dis (2013) 4(8):e776. doi: 10.1038/cddis.2013.293 23949225 PMC3763454

[131] Melko M Douguet D Bensaid M Zongaro S Verheggen C Gecz J. (AF4/FMR2) family of RNA-binding proteins: insights into the molecular pathology of FRAXE intellectual disability. Hum Mol Genet (2011) 20(10):1873–85. doi: 10.1093/hmg/ddr069 21330300

[132] Luo Z Lin C Guest E Garrett AS Mohaghegh N Swanson S. The super elongation complex family of RNA polymerase II elongation factors: gene target specificity and transcriptional output. Mol Cell Biol (2012) 32(13):2608–17. doi: 10.1128/MCB.00182-12 PMC3434493 22547686

[133] Wittamer V Franssen JD Vulcano M Mirjolet JF Le Poul E Migeotte I. Specific recruitment of antigen-presenting cells by chemerin, a novel processed ligand from human inflammatory fluids. J Exp Med (2003) 198(7):977–85. doi: 10.1084/jem.20030382 PMC2194212 14530373

[134] Ernst MC Sinal CJ. Chemerin: at the crossroads of inflammation and obesity. Trends Endocrinol Metab (2010) 21(11):660–7. doi: 10.1016/j.tem.2010.08.001 20817486

[135] Pereira SS Monteiro MP Costa MM Ferreira J Alves MG Oliveira PF. MAPK/ERK pathway inhibition is a promising treatment target for adrenocortical tumors. J Cell Biochem (2019) 120(1):894–906. doi: 10.1002/jcb.27451 30256438

[136] Penny MK Lerario AM Basham KJ Chukkapalli S Mohan DR LaPensee C. Targeting oncogenic Wnt/beta-catenin signaling in adrenocortical carcinoma disrupts ECM expression and impairs tumor growth. Cancers (Basel) (2023) 15(14):3559. doi: 10.3390/cancers15143559 37509222 PMC10377252

[137] Bueno AC More CB Marrero-Gutierrez J de Almeida ESD Leal LF Montaldi AP. Vitamin D receptor activation is a feasible therapeutic target to impair adrenocortical tumorigenesis. Mol Cell Endocrinol (2022) 558:111757. doi: 10.1016/j.mce.2022.111757 36049598

[138] Hadjadj D Kim SJ Denecker T Ben DL Cadoret JC Maric C. A hypothesis-driven approach identifies CDK4 and CDK6 inhibitors as candidate drugs for treatments of adrenocortical carcinomas. Aging (Albany NY) (2017) 9(12):2695–716. doi: 10.18632/aging.101356 PMC5764399 29283884

[139] Rubin B Pilon C Pezzani R Rebellato A Fallo F. The effects of mitotane and 1alpha,25-dihydroxyvitamin D(3) on Wnt/beta-catenin signaling in human adrenocortical carcinoma cells. J Endocrinol Invest (2020) 43(3):357–67. doi: 10.1007/s40618-019-01127-1 31587178

[140] Bertazza L Barollo S Mari ME Faccio I Zorzan M Redaelli M. Biological effects of EF24, a curcumin derivative, alone or combined with mitotane in adrenocortical tumor cell lines. Molecules (2019) 24(12):2202. doi: 10.3390/molecules24122202 31212829 PMC6630722

[141] Fragni M Fiorentini C Rossini E Fisogni S Vezzoli S Bonini SA. *In vitro* antitumor activity of progesterone in human adrenocortical carcinoma. Endocrine (2019) 63(3):592–601. doi: 10.1007/s12020-018-1795-x 30367443

[142] Hui W Liu S Zheng J Fang Z Ding Q Feng C. Nutlin-3a as a novel anticancer agent for adrenocortical carcinoma with CTNNB1 mutation. Cancer Med (2018) 7(4):1440–9. doi: 10.1002/cam4.1431 PMC5911589 29532999

[143] Zhu Y Wang M Zhao X Zhang L Wu Y Wang B. Rottlerin as a novel chemotherapy agent for adrenocortical carcinoma. Oncotarget (2017) 8(14):22825–34. doi: 10.18632/oncotarget.15221 PMC5410265 28423559

[144] Fiorentini C Fragni M Perego P Vezzoli S Bonini SA Tortoreto M. Antisecretive and antitumor activity of abiraterone acetate in human adrenocortical cancer: A preclinical study. J Clin Endocrinol Metab (2016) 101(12):4594–602. doi: 10.1210/jc.2016-2414 27626976

[145] Pagano E Romano B Izzo AA Borrelli F. The clinical efficacy of curcumin-containing nutraceuticals: An overview of systematic reviews. Pharmacol Res (2018) 134:79–91. doi: 10.1016/j.phrs.2018.06.007 29890252

[146] Mosley CA Liotta DC Snyder JP. Highly active anticancer curcumin analogues. Adv Exp Med Biol (2007) 595:77–103. doi: 10.1007/978-0-387-46401-5_2 17569206

[147] Unlu A Nayir E Dogukan KM Kirca O Ozdogan M. Curcumin (Turmeric) and cancer. J BUON (2016) 21(5):1050–60. 27837604

[148] Subramaniam D May R Sureban SM Lee KB George R Kuppusamy P. Diphenyl difluoroketone: a curcumin derivative with potent in *vivo* anticancer activity. Cancer Res (2008) 68(6):1962–9. doi: 10.1158/0008-5472.CAN-07-6011 18339878

[149] Lodish M. Genetics of adrenocortical development and tumors. Endocrinol Metab Clin North Am (2017) 46(2):419–33. doi: 10.1016/j.ecl.2017.01.007 PMC5424622 28476230

[150] Gaujoux S Tissier F Groussin L Libe R Ragazzon B Launay P. Wnt/beta-catenin and 3’,5’-cyclic adenosine 5’-monophosphate/protein kinase A signaling pathways alterations and somatic beta-catenin gene mutations in the progression of adrenocortical tumors. J Clin Endocrinol Metab (2008) 93(10):4135–40. doi: 10.1210/jc.2008-0631 18647815

[151] Lu W Lin C Li Y. Rottlerin induces Wnt co-receptor LRP6 degradation and suppresses both Wnt/beta-catenin and mTORC1 signaling in prostate and breast cancer cells. Cell Signal (2014) 26(6):1303–9. doi: 10.1016/j.cellsig.2014.02.018 PMC4006155 24607787

[152] Ryan CJ Smith MR Fizazi K Saad F Mulders PF Sternberg CN. Abiraterone acetate plus prednisone versus placebo plus prednisone in chemotherapy-naive men with metastatic castration-resistant prostate cancer (COU-AA-302): final overall survival analysis of a randomised, double-blind, placebo-controlled phase 3 study. Lancet Oncol (2015) 16(2):152–60. doi: 10.1016/S1470-2045(14)71205-7 25601341

[153] Fiskus W Sharma S Saha S Shah B Devaraj SG Sun B. Pre-clinical efficacy of combined therapy with novel beta-catenin antagonist BC2059 and histone deacetylase inhibitor against AML cells. Leukemia (2015) 29(6):1267–78. doi: 10.1038/leu.2014.340 PMC4456205 25482131

[154] Nomura M Rainusso N Lee YC Dawson B Coarfa C Han R. Tegavivint and the beta-catenin/ALDH axis in chemotherapy-resistant and metastatic osteosarcoma. J Natl Cancer Inst (2019) 111(11):1216–27. doi: 10.1093/jnci/djz026 PMC6855956 30793158

[155] Li J Wang CY. TBL1-TBLR1 and beta-catenin recruit each other to Wnt target-gene promoter for transcription activation and oncogenesis. Nat Cell Biol (2008) 10(2):160–9. doi: 10.1038/ncb1684 18193033

[156] Dimitrova YN Li J Lee YT Rios-Esteves J Friedman DB Choi HJ. Direct ubiquitination of beta-catenin by Siah-1 and regulation by the exchange factor TBL1. J Biol Chem (2010) 285(18):13507–16. doi: 10.1074/jbc.M109.049411 PMC2859511 20181957

[157] Margalef P Kotsantis P Borel V Bellelli R Panier S Boulton SJ. Stabilization of reversed replication forks by telomerase drives telomere catastrophe. Cell (2018) 172(3):439–453.e14. doi: 10.1016/j.cell.2017.11.047 29290468 PMC5786504

[158] Yuan H Wu Y Wang J Qin X Huang Y Yan L. Synergistic effects of telomerase reverse transcriptase and regulator of telomere elongation helicase 1 on aggressiveness and outcomes in adrenocortical carcinoma. Biomed Pharmacother. (2022) 149:112796. doi: 10.1016/j.biopha.2022.112796 35279598

[159] Infante JR Cassier PA Gerecitano JF Witteveen PO Chugh R Ribrag V. A phase I study of the cyclin-dependent kinase 4/6 inhibitor ribociclib (LEE011) in patients with advanced solid tumors and lymphomas. Clin Cancer Res (2016) 22(23):5696–705. doi: 10.1158/1078-0432.CCR-16-1248 PMC5621377 27542767

